# Supplementary figures and images for: Novel Insights into the Nature of Intraspecific Genome Size Diversity in Cannabis sativa L
Source: Plants (Basel). 2022 Oct 16;11(20):2736. doi: 10.3390/plants11202736 (PMC9607409; doi:10.3390/plants11202736)

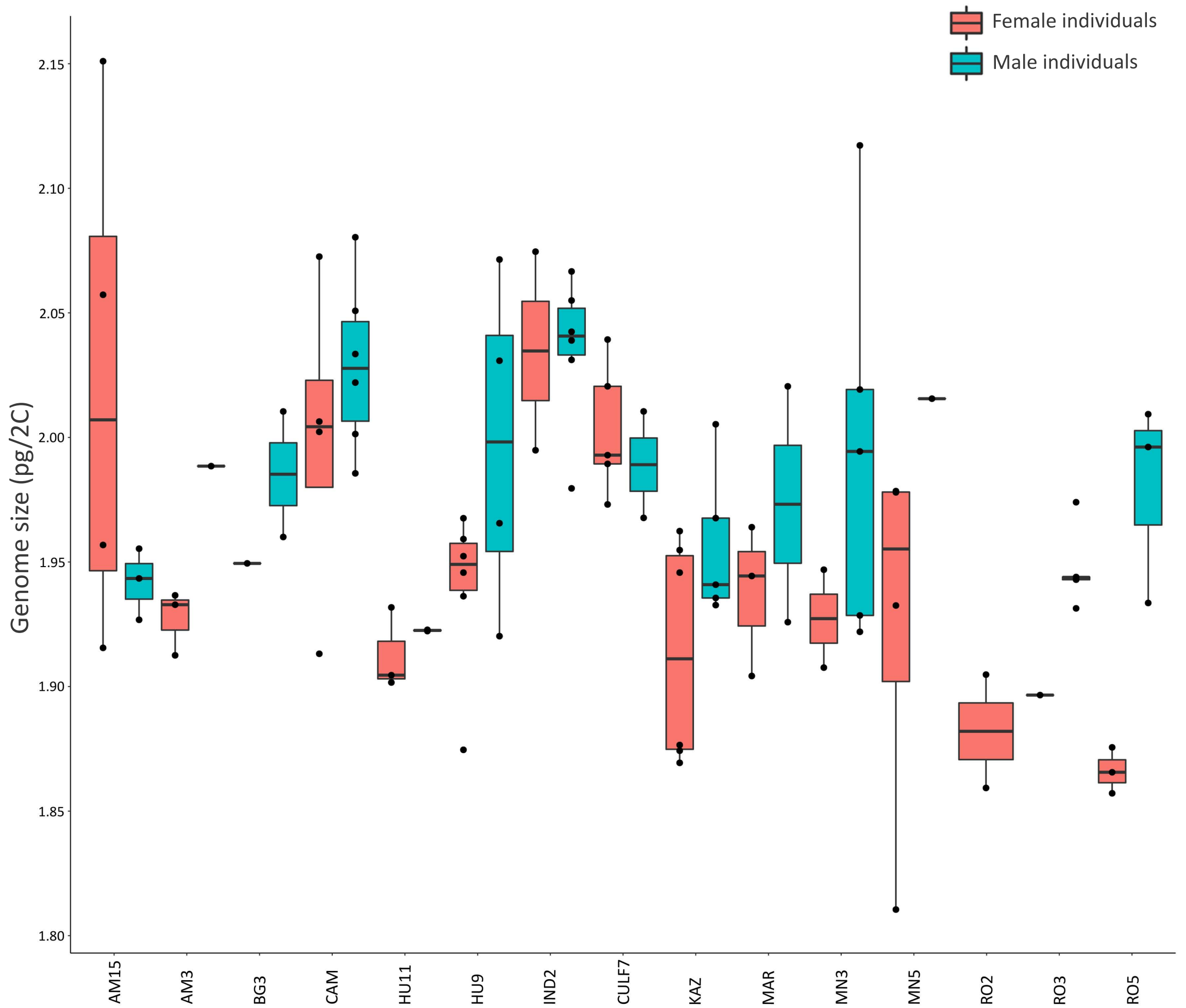

Supplement: Supplementary file 1 [file plants-11-02736-s001.zip › FigureS1.pdf]
